# 

**Published:** 1856-08

**Authors:** 


### PUBLICATIONS RECEIVED.

We have received the two first numbers of the "Louisville Review, a bi-monthly Journal of Practical Medicine and Surgery." Edited by S. D. Gross, M. D., Professor of Surgery in the Jefferson Medical College of Philadelphia, and J. G. Richardson, M. D., Louisville, Kentucky; a valuable Journal, it is unnecessary for us to say, having announced the name of the Senior Editor. It affords us pleasure to exchange.

We have also received "A catalogue of all the graduates of the Jefferson Medical College, of Philadelphia, with announcement for 1856-57," exhibiting a total of 3,597; the number in attendance, at the last session, being 510, and of those graduating, 215.

Also, the "Twenty-fifth annual announcement of the Medical College of Georgia, at Augusta," with a catalogue of the graduates up to the commencement, held March, 1856. Also, the announcement of the St. Louis Medical College for 1856-7, which we regret to say has been mislaid, and we are therefore unable to give their statistics; we recollect, however, one feature, which attracted our attention in a hasty glance, of which we highly approve, (a similar feature being observed in the announcement of the New Orleans School of Medicine, noticed in our last number,) and hope soon to see it ingrafted upon every medical Institution in the land: it is this, under the head of requirements for the Degree of Doctor of Medicine, it is stated that "The candidate must, also, at the time of receiving the Degree, acknowledge the right of the Faculty to revoke it, if they shall, at any time become satisfied, that he has engaged in irregular or unprofessional practices."

What more fit and proper than that the individual who basely deserts his colors, should be branded with the name of *traitor?*

What more fit and proper than that credentials, intended for high and holy uses, should be withdrawn, when perverted to base and dishonorable ends?

We also find upon our table a pamphlet of some 25 pages, being a reprint from the Louisville Review, of "Remarks on Vesico-Vaginal Fistula, with an account of a *new mode of suture,* and seven successful operations by N. Bozeman, M. D., of Montgomery, Alabama."

Dr. Bozeman, in this article, notices the respective operations of Drs. Mettauer, of Virginia, Hayward, of Boston, Pancoast, of Philadelphia, and J. Marion Sims, late of Montgomery, now of New York, considering the method of the latter gentleman by the "clamp suture," as the only one worthy of much discussion, or likely to be adopted in this country, at least, to which, however, he considers that there are a number of objections, and recommends as a substitute, the "button suture," invented by himself, which he describes, and before detailing the particular cases operated upon, sums up the advantages of the new operation by the following remarks: "My experience with the 'button suture,' as detailed in the history of the seven operations appended to this paper, has, I think, established its superiority to the clamp suture in the following particulars:

"1st. It is simple in its construction, and applicable to a greater number of cases.

"2d. It affords complete protection and perfect rest to the approximated edges of the fistule.

"3d. If two fistulous openings exist, one or both may be closed, at the same sitting, according to the inclination of the operator or patient, without reference to the condition of the parts.

"4th. The introduction of the sutures does not demand the same exactness in regard to the position of the points.

"5th. The independent action of each suture renders parallelism unnecessary, and thus gives the operator the liberty of introducing them in whatever direction may best suit his purpose."

---

☞ The Professor of Surgery hereby acknowledges the reception of a most admirable specimen of Dry Gangrene, presented by Chas. W. Ashby, M. D., of Alexandria, Va., accompanying which is also an Obstetrical specimen of value for the *museum.* To Dr. Ashby our warmest thanks are due for this and other substantial evidences of friendly interest in the success of the Atlanta Medical College and Journal.

We have also received (too late for the present number) from the same source, an interesting history of the case from which the Gangrenous foot was obtained, with remarks upon the disease and the propriety of an operation, which will appear in the September number.

---

### TO THOSE WHOM IT MAY CONCERN.

Taste, as well as policy, will keep us out of a controversy with *shirt-tail medicine.*

### TO CORRESPONDENTS.

☞ All Communications pertaining to the Editorial Department of the Journal, should be addressed to the Editors.

☞ All Communications, having reference to the Subscription, the Advertising or the Financial Department, should be addressed to the Treasurer, J. G. WESTMORELAND, M. D., Dean of the Faculty of the Atlanta Medical College.

☞ Messrs. A. TILDEN and W. H. PHILLPOT are authorized Agents for this Journal.

